# Effects of Glyceryl Monolaurate on Production Performance, Egg Quality, Oviduct Cytokines and Intestinal Microflora of 66 Weeks Old Laying Hens

**DOI:** 10.3390/ani13020215

**Published:** 2023-01-06

**Authors:** Zhenchuan Cui, Ruiqiang Zhang, Bing Dai, Chunsheng Fu, Guiling Zhao, Yinglei Xu, Caimei Yang

**Affiliations:** College of Animal Science and Technology, College of Animal Medicine, Zhejiang Agriculture & Forestry University, Hangzhou 311300, China

**Keywords:** glycerol monolaurate, laying rate, egg quality, oviduct cytokines, intestinal villi morphology, intestinal microflora

## Abstract

**Simple Summary:**

It has been proved that glyceryl monolaurate is a safe and green additive in food and feed. The purpose of this experiment was to study the potential application value of glyceryl monolaurate in the diet of laying hens in the late laying period. Our results indicate that the dietary supplementation of glyceryl monolaurate could significantly improve laying performance and egg quality, reducing inflammation, promoting immune function and intestinal barrier function as well as regulating the intestinal flora of laying hens.

**Abstract:**

The principal purpose of this research was to study the effects of glycerol monolaurate (GML) on the production performance; egg quality; health state of the oviduct, ovary and ileum; and gut microbiota of laying hens in the later stage. The laying hens were randomly assigned to two groups: a control group and an experiment group, for which 1000 mg/kg of GML was added to a control diet. The results showed that GML increased the laying rate, average egg weight, albumen height, yolk color and Haugh unit and decreased the feed conversion ratio and defective eggs (*p* < 0.05). GML increased the intestinal villi height and the ratio of villus height to crypt depth (*p* < 0.05). Moreover, GML improved the contents of cytokines in the oviduct, ovary and ileum mucosa; ameliorated the expression of *TLR2*, *TLR4*, *MyD88*, *IL-4*, *IL-1β* and *TNF-α*; and increased the expression of *Occludin* and *Muc-2* in the ileal mucosa. The supplementation of GML increased the volatile fatty acids in the cecal contents, such as acetic acid and propionic acid, and up-regulated *Bacteroides* (*p* < 0.01) and *Alistipes* (*p* < 0.05) richness in the cecal contents. In summary, GML improved production performance, egg quality and immunity; ameliorated the health status of the oviduct, ovary and ileum; enhanced the intestinal barrier function; improved the content of intestinal volatile fatty acids; and regulated the abundance of cecal flora.

## 1. Introduction

Laying hens in the late egg-laying period have low laying rate and poor production performance [1]. This is a recurrent problem in commercial egg layer flocks, which leads to a decline in egg production, increase in mortality and consequent economic losses, as well as decreased animal welfare [2,3,4]. In the poultry industry, the oviduct provides an environment that produces all the structural components of the egg [5]. Therefore, ameliorating the health status of the oviduct may improve the production performance of layers in the later period of laying.

It has been reported that medium-chain fatty acids (MCFAs; C8-C12) as natural feed additives have a positive impact on growth performance, egg quality and intestinal health and have been proved to be a good substitute for antibiotics in the poultry industry [6]. Glycerol monolaurate (GML), a natural monoglyceride of C12:0, exists in breast milk, and coconut and palm oil and can be used in the feed industry. The U.S. Food and Drug Administration (FDA) approved GML as a food-safe emulsifier which is considered non-toxic in food and health products with a content range of 10 to 2000 mg/kg [7]. Additionally, GML can be formed by a combination of lauric acid and glycerol [8]. Researchers found that GML had positive effects as an antiviral and could enhance immunity, thus reducing the viral incidence rate and improving economic benefits [9]. Moreover, GML could enhance growth performance, reduce the number of parasite oocysts and harmful bacteria, and increase the level of globulin [10]. Lan et al. [11] found that GML could modulate the cecal microbiota and immunity of birds. However, the regulatory mechanisms of GML on the oviduct health of laying hens remain unclear.

In the present study, we speculate that GML has the latent capacity to improve the status of the oviduct and ileum, and the production performance of laying hens. The laying performance, egg quality, intestinal morphology, health status of the oviduct and ileum, and composition and distribution of the intestinal microorganism are compared and analyzed between a control diet group and the control diet group supplemented with GML. 

## 2. Materials and Methods

### 2.1. Animal and Treatment

The animal breeding and treatment procedures during the experiment were approved by the Animal Care and Use Committee of Zhejiang A&F University. A total of 600 Lohmann Grey layers (66 weeks old, provided by Lixin Deqingyuan Agricultural Technology Co., Ltd., Bozhou, China) were randomly assigned to two groups, with 10 replicates per treatment and 30 laying hens in each replicate. Each cage (30 cm × 40 cm × 40 cm) was provided with one nipple drinker for a layer to drink from. Each of the 30 hens constitutes an experimental unit, sharing a common feed-through. A two-week pre-experiment was conducted to ensure that all hens used in this study had the same health status and laying rate. The room temperature was kept between 20 and 25 °C during the experiment. The groups and treatment of the experiment were as follows: The control group layers were fed a control diet (CG, Table 1), and the experiment group layers were fed the control diet supplemented with 1000 mg/kg of glycerol monolaurate (EG). GML (90% purity) was produced and provided by Zhejiang Vegamax Biotechnology Co., Ltd. (Huzhou, China). Hens could feed and drink freely. Sixteen hours of light and eight hours of darkness were provided daily. 

The experiment lasted 28 days. All diets were prepared in accordance with the recommendations of the published NRC (1994) and the Lohmann hen manual. 

### 2.2. Sample Collection

One bird was randomly selected from each replicate of the treatment and humanely euthanized at the end of the trial to collect blood from the carotid artery. The precipitated serum was then separated into 1.5 mL centrifugal tubes and immediately stored at −80 °C for further study. After slaughter, two parts of the oviduct isthmus, the dilated part of the oviduct and ovary segments were collected and were stored in a fresh 4% paraformaldehyde solution and at −80 °C in a refrigerator for further tests. The ileal mucosa was gathered for the detection of inflammatory factors and gene expression. The contents of the cecum were collected with sterile instruments and stored at −80 °C in a refrigerator for the detection of volatile fatty acid (VFA) and the analysis of microflora.

### 2.3. Production Performance

Egg production and feed consumption were recorded every day. The laying rate was obtained by dividing the number of eggs collected by the number of layers. The feed conversion ratio was obtained by dividing the daily feed intake by the daily egg mass output. The number of defective eggs (soft-shell eggs, spotted eggs, misshapen eggs, and pimpled eggs) and broken eggs was recorded.

### 2.4. Egg Quality

At the end of the experiment, two eggs from each cage were randomly collected. The eggs (10 replicates per group, two eggs per cage) were broken and placed on a flat surface, where the Haugh unit, yolk color, height of the albumen and shell strength were measured with an egg quality analyzer (ET-6000, Nabel, Kyoto, Japan). When measuring the thickness of the eggshells, we cleaned them to remove the shell membrane and measured from three different parts of the eggshell (mm) using a spiral micrometer (217-111, Nanjing Sucian Measuring Instrument Co., Ltd., Nanjing, China), and subsequently, the average of the three measurements was calculated.

### 2.5. Inflammatory Cytokines

The concentrations of interferon-γ (IFN-γ) tumor necrosis factor-α (TNF-α), interleukin-1β (IL-1β), interleukin-2 (IL-2), interleukin-6 (IL-6), interleukin-4 (IL-4), and interleukin-10 (IL-10) were detected with commercial ELISA kits (Angle Gene Biological Technology Co., Ltd., Nanjing, China) according to the instruction manual.

### 2.6. Histological Morphology

The ileum samples stored in 4% paraformaldehyde were dehydrated with ethanol and washed with xylene, and the tissue samples were embedded in paraffin. The sliced paraffin was fixed, sectioned, deparaffinized and stained with hematoxylin–eosin. The slides were observed with a Nikon optical microscope (Tokyo, Japan) connected to a computer.

### 2.7. Real-Time Quantitative PCR

The RNA of the oviduct, ovary and ileal mucosa was isolated by Trizol reagent (Takara Bio Inc., Beijing, China). The quality and concentration of extracted total RNA were determined by a Nano-300 microspectrophotometer (No. 1053, Allsheng Co., Ltd., Hangzhou, China) and diluted to the same concentration with DEPC water. The cDNA used in real-time quantitative PCR was obtained by reverse transcription of qualified RNA by a PrimeScript^TM^ RT Master Mix regent kit (Takara, Kusatsu, Japan) with a gDNA eraser. The primer sequences and related information are listed in Table 2 and synthesized by TSINGKE Biological Technology (Hangzhou, China). The relative mRNA levels of the target genes were analyzed using the 2^−∆∆Ct^ method.

### 2.8. Volatile Fatty Acid Analysis

Based on previous studies [12,13], the content of VFA in cecum was determined by Headspace gas chromatography (Agilent Technologies, Wilmington, DE, USA). Briefly, 0.5 g of cecal contents was blended with water. The supernatant was then extracted with syringes after centrifugation, mixed with 25% (m/v, 1:5) phosphorous acid, and filtrated and injected into 2 mL sample loading bottles with a 0.22 μm membrane. An apparatus equipped with a 5183-4711 liner tube and 10 μL Agilent Gold Standard autosampler syringes was used for detection.

### 2.9. Intestinal Microbiota Community

Total microbial DNA was extracted from the cecal contents using the CTAB method. The V3–V4 region of the bacterial 16S rRNA gene was amplified. After amplification and purification, equal molar amounts of amplicons were collected and sequenced. Analysis of relevant indicators was subsequently conducted using the Illumina HiSeq platform Novogene (Beijing, China). Relevant materials were uploaded to NCBI (accession: PRJNA856982).

### 2.10. Statistical Analysis

Data were compiled in Excel 2019 and analyzed with independent sample *t*-tests using SPSS 22.0 (SPSS Inc., Chicago, IL, USA). Data for production performance, egg quality, inflammatory factors and VFA analyses are expressed as values of mean and standard error (SEM). When *p* < 0.05, it is considered that there is a significant difference. Figures were made in Prism, version 8.0 (GraphPad Software Inc., San Diego, CA, USA). 

## 3. Results

### 3.1. Production Performance

As shown in Table 3, diets supplemented with GML significantly increased the average egg weight and the laying rate compared with the control group in the later stages of laying hens (*p* < 0.05). The feed conversion ratio and the defective eggs in the EG were lower than those of eggs in the control group (*p* < 0.05). GML had no obvious effect on the feed intake, broken eggs or the pimpled eggs in the later stages of laying (*p* > 0.05).

As displayed in Table 4, albumen height, Haugh unit and yolk color in the EG were markedly increased (*p* < 0.05). The results in Table 4 indicate that a diet supplemented with GML had no significant effect (*p* > 0.05) on the shell thickness and strength of the eggshells. 

### 3.2. Ileum Morphology

Table 5 shows the histological structure of the ileum. A diet supplemented with GML enhanced the villus height of the ileum compared with that in the CG (*p* < 0.05). Moreover, GML has no obvious effect on crypt depth (*p* > 0.05) compared with those in the CG. Furthermore, the villus height-to-crypt depth ratio was significantly up-regulated by GML treatments in the later stages of laying hens (*p* > 0.05). 

### 3.3. Tissue Inflammatory Factors

The EG laying hens had lower concentrations of IL-2, IL-1β, TNF-α and IFN-γ (Table 6; *p* < 0.05) and a higher level of IL-10 (*p* < 0.01) in their oviduct isthmus than did the control laying hens. No differences in IL-4 and IL-6 levels were found between the CG and EG in the oviduct isthmus (*p* > 0.05). Laying hens in the EG had lower concentrations of IL-2, IL-1β, TNF-α and IFN-γ and higher concentrations of IL-4 and IL-10 during oviduct dilatation than did the control laying hens (*p* < 0.05). In the ovary and ileum mucosa, GML decreased the levels of IL-2, IL-6, IL-1β, TNF-α and IFN-γ and increased the IL-4 and IL-10 levels (*p* < 0.05) of EG laying hens in the later stage.

### 3.4. Relative mRNA Expression of Oviduct, Ovary and Ileum Mucosa Genes

**Figure 1:**
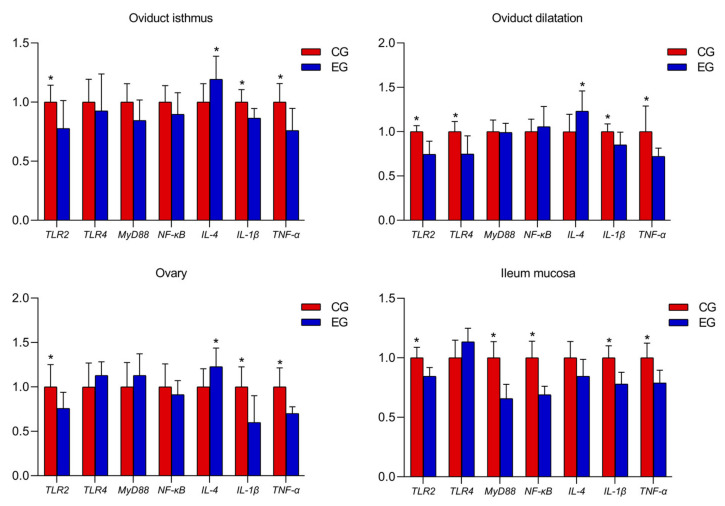
Effects of glyceryl monolaurate on gene expression levels in the oviductal isthmus during oviduct dilatation, and in the ovary and ileum mucosa in laying hens. (*p < 0.05).

GML down-regulated the oviduct isthmus *IL-1β*, *TNF-α* and *TLR2* mRNA expression and up-regulated the *IL-4* expression level compared with the CG (Figure 1; *p* < 0.05). The gene expression levels of *IL-1β*, *TNF-α*, *TLR2* and *TLR4* decreased, and the expression level of *IL-4* increased during oviduct dilatation with GML supplementation compared with the CG (*p* < 0.05). In the ovary, the mRNA expression of *TLR2*, *TLR4*, *IL-1β* and *TNF-α* decreased and the mRNA expression of *IL-4* increased in GML-treated layers (*p* < 0.05).

**Figure 2:**
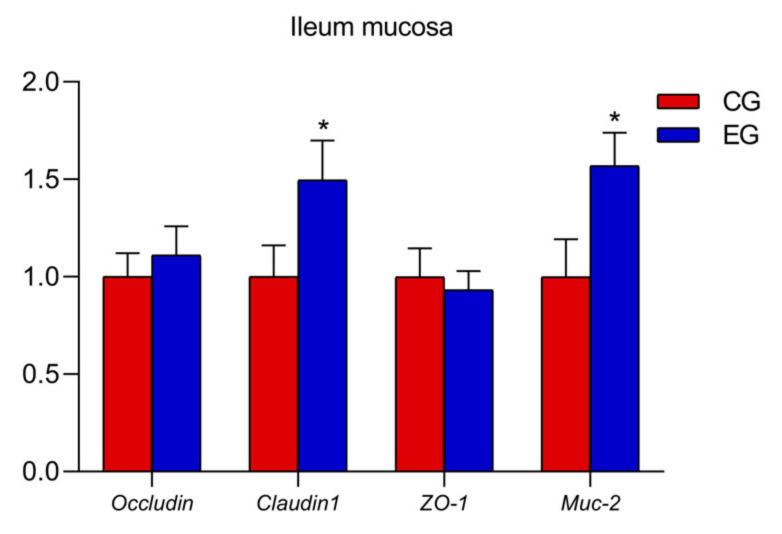
Effects of glyceryl monolaurate on gene expressions related to ileal mucosal barrier in laying hens. (* *p* < 0.05).

In the ileum mucosa, the expression levels of *TLR2*, *MyD88*, *IL-1β* and *TNF-α* decreased (Figure 1; *p* < 0.05) and the mRNA expression *Occludin* and *Muc-2* increased in the EG compared with the CG (Figure 2; *p* < 0.05).

### 3.5. Volatile Fatty Acid Levels in Cecal Contents

As displayed in Table 7, GML significantly increased the levels of acetic acid and propionic acid (*p* < 0.05). There were no obvious differences (*p* > 0.05) in isobutyric, butyrate, isovaleric and valeric acid levels between the CG and EG.

### 3.6. Cecal Microbial Community

**Figure 3:**
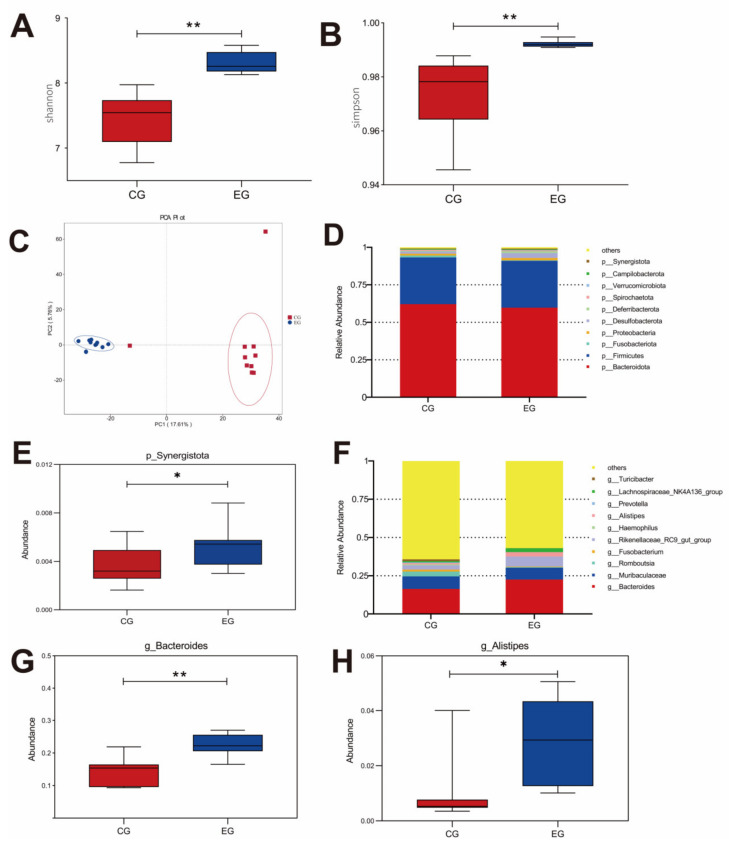
Effects of glyceryl monolaurate on cecal microbiota diversity in laying hens. (**A**) Shannon index. (**B**) Simpson index. (**C**) Principal component analysis (PCA) scatterplot. (**D**,**E**) The The microbiota composition on phylum level. (**F**–**H**) The microbiota composition on genus level. * *p* < 0.05, ** *p* < 0.01.

GML changed the abundance of cecal microbiota in laying hens (Figure 3). The Shannon and Simpson diagrams indicated that diversity was visibly higher in the EG laying hens than that in the CG. According to the PCA (principal component analysis) scatterplot, there were obvious differences in microbiota structure between the CG and EG, and no overlapping clusters were found. The changes in microbial community composition were analyzed on the phylum and genus levels. The major phyla were Bacteroidota, Firmicutes and Fusobacteriota (Figure 3D). Synergistota was increased significantly in the EG (*p* < 0.05). The dominant genera were *Bacteroides*, *Muribaculaceae* and *Romboutsia.* GML up-regulated the richness of *Bacteroides* and *Alistipes* in the EG (*p* < 0.05). 

## 4. Discussion

In recent years, the role played by medium-chain fatty acids in feed additives has attracted much attention. According to previous studies, GML had positive effects in promoting growth and immunity in animals [14]. Liu et al. [14] found that adding GML to feed improved the production performance of layers. In the present study, a diet with added GML resulted in an increase in the laying rate and egg weight and a decrease in the feed conversion ratio, which was a similar result to those of previous reports on the addition of MCFA to improve production performance [11,14]. Dietary GML supplementation effectively affects the metabolic status of laying hens [15,16], and GML is also a good energy supply substance for animals, promoting growth and regulating immunity by acting on intestinal flora and host health and producing beneficial effects [17]. Liu et al. [18] found that GML could enhance production performance by improving serum FSH and LH levels in laying hens.

Eggshell thickness and eggshell strength are two major parameters for evaluating egg quality. Albumen height and the Haugh unit are important indexes with which to assess albumen quality and egg freshness. Yolk color is the most important indicator of yolk quality, because consumers prefer dark yellow yolks [19]. The results of the present study indicated that GML significantly improved egg protein height, egg yolk color and the Haugh unit, and parallel the results of other studies. Liu et al. [14] found that dietary GML supplementation increased eggshell thickness and eggshell strength. GML could also improve the quality and thermal stability of egg protein [20]. The enhancement of yolk color may have been due to the influence of GML on the absorption and utilization of feed lipids and lipid-soluble carotenoid pigments [21]. 

The integrity of the intestinal tract is critical to preventing pathogenic microbial invasion in animals [22]. The VCR reflects a stronger digestion and absorption capacity or more robust intestinal functions [23,24], which may be one of the reasons for the absorption and digestion of feed nutrients and the decrease in the feed conversion ratio [25]. GML has an antibacterial effect that can inhibit pathogenic bacteria and reduce the production of toxins, which tends to make the intestinal state more stable and healthier so as to ensure better digestion and absorption capacity in animals [9,26,27]. This study found that GML supplementation in the diet could markedly increase the intestinal VH and VCR of laying hens, which is consistent with past research on the effects of GML on the intestinal structure of laying hens [14], suggesting that GML ameliorated the intestinal structure of laying hens. These results are in line with the improved production performance of the GML group of laying hens in this study.

*Muc-2* encodes mucin in the gastrointestinal tract, a protective mucosal layer which plays an important role in defending against microbial attacks [28]. Claudins are key transmembrane proteins and form the skeleton of tight junctions (TJs) by promoting intercellular adhesion [29]. Moreover, the ZO (zonula occludens) of cytoplasmic proteins could connect transmembrane proteins, such as claudin and occludin, to the cytoskeleton, and cells with ZO-1 deletion cannot form TJs [30,31]. Researchers found that mice treated with GML up-regulated the gene expression of *ZO-1*, *Muc-2* and *Claudin-1*, indicating that GML had a beneficial effect on intestinal health and mucosal barrier function [32]. In the present study, dietary GML supplementation clearly up-regulated ileum *Occludin* and *Muc-2* mRNA expression. These results illustrated that GML positively affected the intestinal barrier function of laying hens.

It was reported that GML has antibacterial and anti-inflammatory efficacy [32] and plays an immunomodulatory role in interfering with human T cells, which may reduce the production of cytokines induced by T cell receptors [33]. GML could enhance the serum IgM and IgY content in broilers [11]. Dietary treatment with GML reduced IL-1β and TNF-α content and increased the serum IgG level [34]. Similarly, we found that GML ameliorated oviduct, ovary and ileum IL-2, IL-4, IL-6, IL-10, IL-1β, TNF-α and IFN-γ levels, suggesting that GML could regulate the inflammatory response of laying hens and could improve the health of laying hens by stabilizing the balance of cytokines. TNF-α and IFN-γ play detrimental roles by decreasing the epithelial barrier function and increasing mucosal barrier permeability [35]. Other studies have found that fructo oligosaccharides have an anti-inflammatory function and contribute to the recovery of the intestinal morphology of broilers that have been attacked by LPS. This suggests that a reduction in the level of proinflammatory cytokines and the down-regulation of the TLR/NK-κB signal transduction may improve intestinal integrity [36,37].

GML has a positive anti-inflammatory effect and can exert anti-inflammatory effects locally [38]. In the present study, GML reduced the mRNA expression of IL-1β and TNF-α in the oviduct, ovary and ileum. In an experiment on GML in mice, GML down-regulated the mRNA expression of *TLR2* and *MyD88* in the liver and reduced inflammatory responses [34]. NF-κB is an important transcription factor in cells that regulate inflammatory responses by modulating the signaling pathways regulated by innate immune receptors [39]. The effects of GML on mice were similar to those found in this study, but the inflammatory responses were at a lower level in the present study; inflammatory cytokines are not rapidly produced without infection or other stimuli when laying hens are raised under normal conditions [40]. To synthesize the above results, GML may regulate the Toll/MyD88 pathway and improve the immune capacity of laying hens.

Intestinal microbiota is important for intestinal morphological structure and physiological function, which are considered to be closely related to gut health, barrier function and the growth performance of the host [41]. In the present study, the inclusion of GML increased the Shannon and Simpson index, indicating that GML could improve microbial diversity. In addition, the separated PCA revealed that the microbiota compositions of the two groups were different in the present study. Compared with the CG, the amount of Synergistota was greater in the GML group. Previous research found that Synergistota could inhibit the generation of toxins in diets and stabilize the intestinal environment in ruminants [42]. At the genus level, GML increased the *Bacteroides* population, which could ferment carbohydrates to produce VFAs, providing energy for the gut and subsequently promoting growth performance in animals [43,44]. In addition, VFAs can reduce the immune response of host pro-inflammatory factors IL-1β, IL-6 and TNF-α through the TLR4 pathway [45], which is consistent with the results of the tissue inflammatory factors in this study. A previous study reported that *Alistipes* has protective effects against cardiovascular-related diseases, inflammatory bowel disease and neurological diseases [46]. In this study, GML enhanced the abundance of *Alistipes*, suggesting that GML may have a positive impact on immunity by increasing the abundance of *Alistipes* in laying hens.

## 5. Conclusions

In summary, the dietary supplementation of GML could improve production performance and egg quality by ameliorating immunity, intestinal and oviduct cytokines, improving intestinal barrier function and adjusting intestinal microflora, which could develop a new immunomodulator in laying hens.

## Figures and Tables

**Table 1:** Composition and nutrient levels of the control diet (air-dried level).

Ingredients	Content, %	Nutrient Level ^2^	Content
Corn	63.50	ME, MJ/kg ^2^	11.41
Soybean meal	22.00	CP, % ^3^	15.91
Soybean oil	0.30	Ca, % ^3^	4.06
Bran	5.00	TP, % ^3^	0.48
Limestone	7.90	Met, % ^3^	0.32
NaCl	0.30	Lys, % ^3^	0.60
Premixe ^1^	1.00	Thr, % ^3^	0.64
Total	100.00		

^1^ Provided per kg of premix: vitamin A, 10,000 IU; vitamin D3, 1000 IU; vitamin E, 11 IU; vitamin K, 1.50 mg; vitamin B1, 1.2 mg; vitamin B2, 6.5 mg; vitamin B5, 10 mg; vitamin B6, 6 mg; vitamin B12, 0.012 mg; choline, 500 mg; pantothenic, 7 mg; niacin, 40 mg; folic acid, 0.55 mg; Zn, 75 mg; Fe, 80 mg; Mn, 60 mg; Cu, 10 mg; Se, 0.3 mg; I, 0.6 mg. ^2^ Metabolizable energy is calculated; other values are measured.^3^ The numbers were analyzed values.

**Table 2:** Nucleotide sequences for real-time PCR primers.

Name	Position	Primer Sequence, 5′→3′	Product Size (bp)	Access No.
β-Actin	Forward	TGCTGTGTTCCCATCTATCG	150	NM_205518
	Reverse	TTGGTGACAATACCGTGTTCA		
TLR2	Forward	CATTCACCATGAGGCAGGGATAG	157	NM_001396826.1
	Reverse	GGTGCAGATCAAGGACACTAGGA		
TLR4	Forward	TGACCTACCCATCGGACACT	171	NM_001030693.2
	Reverse	CTCAGGGCATCAAGGTCTCC		
MyD88	Forward	GATGATCCGTATGGGCATGGA	170	NM_001030962.4
	Reverse	ATGGACCACACACACGTTCC		
NF-κB	Forward	TGCCTTTTGCTTGAGGGTGATG	100	XM_015285418.2
	Reverse	CTGCCAGTTTTGTGAAGCCC		
IL-1β	Forward	CGACATCAACCAGAAGTGCTT	298	XM_015297469.1
	Reverse	GTCCAGGCGGTAGAAGATGA		
IL-4	Forward	AGCACTGCCACAAGAACCT	160	NM_001398460.1
	Reverse	GCTAGTTGGTGGAAGAAGGTAC		
TNF-α	Forward	CCGTAGTGCTGTTCTATGACCG	125	NM_204267.2
	Reverse	GTTCCACATCTTTCAGAGCATCAA		
Muc-2	Forward	ATTGTGGTAACACCAACATTCATC	135	XM_0012334581.3
	Reverse	CTTTATAATGTCAGCACCAACTTCTC		
ZO-1	Forward	GGATGTTTATTTGGGCGGCT	153	XM_015278980.2
	Reverse	CCATTGTTGCACTCTTGCCG		
Claudin-1	Forward	CACACCCGTTAACACCAGATTT	159	NM_001013611.2
	Reverse	GAGGGGGCATTTTTGGGGTA		
Occludin	Forward	AGCCCTCAATACCAGGATGTG	125	NM_205128.1
	Reverse	CGCTTGATGTGGAAGAGCTTG		

**Table 3:** Effects of glyceryl monolaurate on the production performance of laying hens.

Items	Control Group	Glyceryl Monolaurate Group	*p*-Value
Average egg weight, g	58.66 ± 0.75	60.97 ± 0.54	0.024
Laying rate, %	83.38 ± 0.59	85.40 ± 0.51	0.019
Feed intake, g	129.27 ± 1.79	130.44 ± 1.20	0.595
Feed conversion ratio, g/g	2.20 ± 0.02	2.14 ± 0.02	0.038
Defective eggs, %	8.75 ± 0.17	8.02 ± 0.19	0.011
Broken eggs, %	0.78 ± 0.04	0.83 ± 0.03	0.358
Pimpled eggs, %	3.27 ± 0.05	3.12 ± 0.09	0.161

The mean ± SEM represents results from 10 replicates per treatment.

**Table 4:** Effects of glyceryl monolaurate on the egg quality of laying hens.

Items	Control Group	Glyceryl Monolaurate Group	*p*-Value
Shell strength, kg·f/m^2^	5.27 ± 0.25	5.39 ± 0.15	0.676
Shell thickness, mm	0.38 ± 0.01	0.39 ± 0.01	0.365
Albumen height, mm	6.09 ± 0.04	6.28 ± 0.07	0.040
Yolk color	9.70 ± 0.15	10.30 ± 0.21	0.036
Haugh unit	78.55 ± 0.54	80.32 ± 0.57	0.037

The mean ± SEM represents results from 10 replicates per treatment.

**Table 5:** Effects of glyceryl monolaurate on the intestinal morphology of laying hens.

Items	Control Group	Glyceryl Monolaurate Group	*p*-Value
Villus height, μm	627.79 ± 21.49	703.82 ± 24.33	0.031
Crypt depth, μm	144.91 ± 8.60	147.47 ± 7.55	0.826
VCR, μm/μm	4.39 ± 0.12	4.81 ± 0.12	0.023

VCR, villus height-to-crypt depth ratio. The mean ± SEM represents results from 10 replicates per treatment.

**Table 6:** Effects of glyceryl monolaurate on the contents of inflammatory cytokines in oviduct, ovary and ileum mucosa of laying hens.

Items	Control Group	Glyceryl Monolaurate Group	*p*-Value
Oviduct isthmus			
IL-2, pg/mg protein	5.65 ± 0.30	5.17 ± 0.35	0.016
IL-4, pg/mg protein	140.31 ± 13.64	155.80 ± 16.78	0.083
IL-6, pg/mg protein	255.51 ± 15.52	237.13 ± 20.98	0.087
IL-10, ng/g protein	25.91 ± 1.52	28.99 ± 2.69	0.022
IL-1β, pg/mg protein	94.72 ± 3.78	88.12 ± 5.12	0.018
TNF-α, ng/g protein	34.15 ± 3.23	30.77 ± 2.01	0.036
IFN-γ, pg/mg protein	377.73 ± 11.66	347.53 ± 20.17	0.007
Oviduct dilatation			
IL-2, pg/mg protein	5.89 ± 0.37	5.33 ± 0.35	0.012
IL-4, pg/mg protein	131.31 ± 7.41	147.61 ± 10.77	0.006
IL-6, pg/mg protein	265.78 ± 18.43	251.45 ± 25.48	0.251
IL-10, ng/g protein	25.01 ± 2.43	28.27 ± 2.43	0.027
IL-1β, pg/mg protein	100.08 ± 1.78	94.25 ± 5.37	0.028
TNF-α, ng/g protein	37.82 ± 2.99	34.44 ± 2.40	0.039
IFN-γ, pg/mg protein	385.31 ± 11.67	361.85 ± 22.09	0.034
Ovary			
IL-2, pg/mg protein	4.15 ± 0.12	3.65 ± 0.44	0.023
IL-4, pg/mg protein	171.81 ± 11.81	187.04 ± 11.24	0.029
IL-6, pg/mg protein	213.61 ± 13.72	191.09 ± 9.58	0.004
IL-10, ng/g protein	35.46 ± 1.55	37.80 ± 1.28	0.010
IL-1β, pg/mg protein	67.87 ± 3.57	63.33 ± 3.94	0.043
TNF-α, ng/g protein	26.58 ± 1.52	24.40 ± 2.01	0.041
IFN-γ, pg/mg protein	323.79 ± 12.06	308.60 ± 10.21	0.026

The mean ± SEM represents results from 10 replicates per treatment.

**Table 7:** Effects of glyceryl monolaurate on volatile fatty acid of cecal contents in laying hens.

Items	Control Group	Glyceryl Monolaurate Group	*p*-Value
Acetic acid, μmol/g	47.70 ± 10.44	59.08 ± 10.48	0.026
Propionic acid, μmol/g	12.75 ± 3.07	15.75 ± 2.97	0.031
Isobutyric acid, μmol/g	1.51 ± 0.28	1.45 ± 0.47	0.691
Butyric acid, μmol/g	5.65 ± 1.82	6.04 ± 2.28	0.678
Isovaleric acid, μmol/g	1.18 ± 0.27	1.23 ± 0.46	0.795
Valeric acid, μmol/g	1.22 ± 0.29	1.16 ± 0.23	0.596

The mean ± SEM represents results from 10 replicates per treatment.

## Data Availability

The data presented in this study are available from the corresponding author upon request.
